# Resolving complex chromosome structures during meiosis: versatile deployment of Smc5/6

**DOI:** 10.1007/s00412-015-0518-9

**Published:** 2015-05-07

**Authors:** Dideke E. Verver, Grace H. Hwang, Philip W. Jordan, Geert Hamer

**Affiliations:** Center for Reproductive Medicine, Women’s and Children’s Hospital, Academic Medical Center, University of Amsterdam, Amsterdam, The Netherlands; Department of Biochemistry and Molecular Biology, Johns Hopkins University Bloomberg School of Public Health, Baltimore, MD USA

**Keywords:** Structural maintenance of chromosomes, Smc5/6, Meiosis, Meiotic recombination, DNA repair

## Abstract

The Smc5/6 complex, along with cohesin and condensin, is a member of the structural maintenance of chromosome (SMC) family, large ring-like protein complexes that are essential for chromatin structure and function. Thanks to numerous studies of the mitotic cell cycle, Smc5/6 has been implicated to have roles in homologous recombination, restart of stalled replication forks, maintenance of ribosomal DNA (rDNA) and heterochromatin, telomerase-independent telomere elongation, and regulation of chromosome topology. The nature of these functions implies that the Smc5/6 complex also contributes to the profound chromatin changes, including meiotic recombination, that characterize meiosis. Only recently, studies in diverse model organisms have focused on the potential meiotic roles of the Smc5/6 complex. Indeed, Smc5/6 appears to be essential for meiotic recombination. However, due to both the complexity of the process of meiosis and the versatility of the Smc5/6 complex, many additional meiotic functions have been described. In this review, we provide a clear overview of the multiple functions found so far for the Smc5/6 complex in meiosis. Additionally, we compare these meiotic functions with the known mitotic functions in an attempt to find a common denominator and thereby create clarity in the field of Smc5/6 research.

## Smc5/6 complex structure

**Fig. 1:**
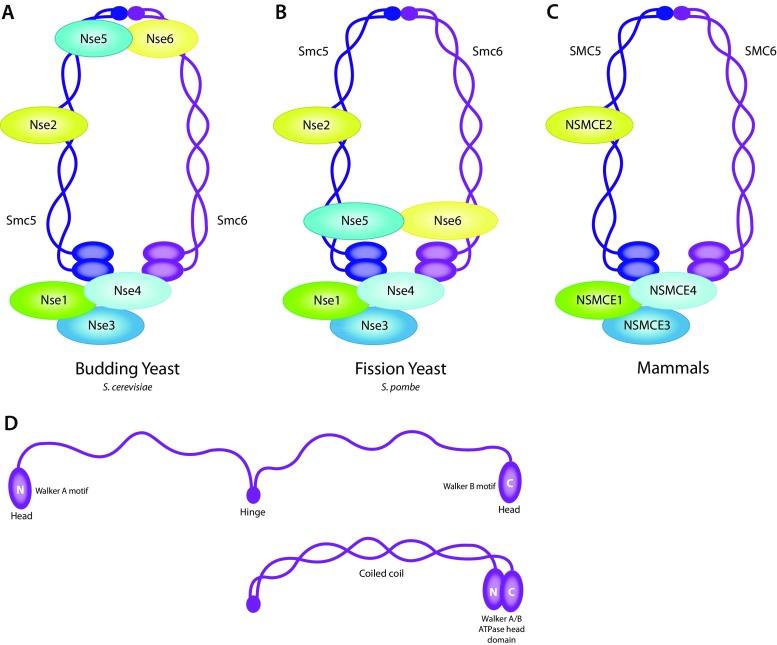
Structure and composition of Smc5/6 complex. Conserved from yeast to humans, Smc5 and Smc6 fold and interact at their central hinge domains. Through the coiled-coil stretch, the N- and C-termini are brought in close proximity creating an ATPase domain. The ring-like structure is closed by several non-SMC elements (Nse1, Nse3, and Nse4). In addition, the SUMO ligase Nse2 is bound to the coiled-coil region of Smc5. Nse5 and Nse6 are located at the hinge domain in budding yeast (**a**), at the ATPase domain in fission yeast (**b**), but homologs have not been identified in mammals (**c**). **d** Smc5 and Smc6 each contain an extensive coiled-coil domain that folds back on itself at a central hinge domain, juxtaposing the terminal head domains to form an ATP-binding and ATP-hydrolysis site

The Smc5/6 complex is a member of the structural maintenance of chromosome (SMC) family, along with cohesin and condensin. The Smc5/6 complex is proposed to have the characteristic ring-like structure of the SMC family in which each SMC complex is comprised of two SMC proteins forming a heterodimer and multiple non-SMC elements (reviewed in (Jeppsson et al. 2014b)). The Smc5/6 complex is comprised of Smc5, Smc6, and several non-SMC elements of which Nse1-4 are conserved from yeast (Duan et al. 2009; Hazbun et al. 2003; Pebernard et al. 2006; Zhao and Blobel 2005) (Fig. 1a, b) to mammals (De Piccoli et al. 2009; Taylor et al. 2008) (Fig. 1c). When referring to the Smc5/6 complex genes or proteins in general, we will use yeast nomenclature. When referring to a specific organism, or data obtained using a specific organism, we will use the specific nomenclature of that organism, e.g., NSMCE1 for the mammalian ortholog of Nse1. The SMC proteins have an extensive coiled-coil domain interrupted by a hinge domain that folds each SMC back on itself. The two globular C and N terminal ends are juxtaposed to form an ATP-binding and ATP-hydrolysis site (Fig. 1d). To form a closed-ring structure, the ATPase domains are bridged together by non-SMC elements, while the SMC proteins associate tightly through their hinge regions (reviewed in (Jeppsson et al. 2014b)).

In vitro assays using purified fission yeast proteins have shown that Nse1 binds to Nse3, and both Nse1 and Nse3 bind to Nse4 (Palecek et al. 2006; Pebernard et al. 2008). Nse1 contains a RING-finger domain, common to ubiquitin E3 ligases (Fujioka et al. 2002; McDonald et al. 2003; Potts 2009), and Nse3 contains a MAGE (melanoma-associated antigen gene) domain (Pebernard et al. 2004). It has been shown that human NSMCE3 enhances the E3 ubiquitin ligase of NSMCE1 in vitro (Doyle et al. 2010). Nse2 (also referred to as Mms21) is bound to Smc5, contains a SP-RING domain (McDonald et al. 2003; Pebernard et al. 2004), and functions as an E3 small ubiquitin-related modifier (SUMO) ligase (Andrews et al. 2005; Potts and Yu 2007; Zhao and Blobel 2005). Nse4 is a α-kleisin subunit which bridges the ATPase head domains of Smc5 and Smc6 (Palecek et al. 2006). Nse5 and Nse6 are also Smc5/6 components in budding and fission yeast, although homologs in other organisms have not been elucidated. In budding yeast, Nse5 and Nse6 associate with the hinge region (Fig. 1a) (Duan et al. 2009). In fission yeast, Nse5 and Nse6 associate with the head domains (Fig. 1b), which may enhance the stability of the complex (Pebernard et al. 2006).

## Smc5/6 in mitotic cells

In somatic cells, the Smc5/6 complex is involved in several processes required to maintain genomic stability. Mechanistically, these processes involve regulation of specific factors required for homologous recombination (HR) pathways. All these processes, including DNA replication, HR-mediated DNA double strand break (DSB) repair, correct chromosome topology and, eventually, proper metaphase conformation, are also essential for successful meiosis.

### Smc5/6 and stalled replication forks

Smc5/6 is required for maintaining replication fork stability and the restart of stalled replication. In budding yeast, the absence of Nse2 SUMO ligase activity results in Rad51-dependent X-shaped HR intermediates or aberrant joint molecules (JMs) accumulating at stalled replication forks (Bermudez-Lopez et al. 2010; Branzei et al. 2006). The Smc5/6 complex functions with Sgs1, a homolog of the Bloom syndrome helicase (BLM), to inhibit the accumulation of these abnormal intermediates. It is possible that this function is conserved in humans, as hypomorphic mutations that lead to the loss of the NSMCE2 SP-RING domain result in delayed recovery from replication stress and a reduction in BLM foci (Payne et al. 2014). These defects result in chromosome bridges and missegregation during the metaphase to anaphase transition. In budding yeast, Smc5/6 has been shown to interact and restrain the replication regression activity of Mph1 helicase, an ortholog of human FANCM, which is required for replication fork repair but can also lead to accumulation of JMs (Xue et al. 2014).

In fission yeast, similar JMs accumulate at the collapsed replication forks in *smc6* mutants, correlating with chromosome missegregation (Ampatzidou et al. 2006). Smc5/6 is required for the loading of Rpa and Rad52 onto stalled replication forks in order for the fork to maintain a recombination-competent conformation (Irmisch et al. 2009). Overexpression of Brc1, a BRCA C-terminal (BRCT) motif protein, rescues the replication-arresting defect of a Smc6 hypomorphic mutant (Lee et al. 2007; Sheedy et al. 2005; Verkade et al. 1999). Because this rescue is dependent on Brc1-mediated promotion of a post-replicative repair pathway and the function of structure-specific endonucleases Slx1/4 and Mus81/Eme1 that resolve the accumulated JMs, Smc5/6 complex may be required to prevent the formation of replication stress-induced JMs and/or assist in their resolution.

### Facilitating homologous recombination

Numerous studies using mammalian, plant, budding yeast, and fission yeast cells have indicated that Smc5/6 functions in the homologous recombination pathway (Ampatzidou et al. 2006; Cost and Cozzarelli 2006; Lehmann et al. 1995; McDonald et al. 2003; Mengiste et al. 1999; Pebernard et al. 2006; Stephan et al. 2011; Torres-Rosell et al. 2005a, b; Watanabe et al. 2009).

In budding yeast and human cells, Smc5/6 and cohesin are recruited to DSBs to promote repair via sister chromatid recombination (De Piccoli et al. 2006; Lindroos et al. 2006; Potts et al. 2006; Strom et al. 2004; Unal et al. 2004; Wu and Yu 2012). Although Smc5/6 and cohesin complexes are recruited to DSBs independently, Nse2-mediated sumoylation of the α-kleisin subunit of cohesin, Scc1, is required to ensure proficient sister chromatid recombination (McAleenan et al. 2012; Wu and Yu 2012). In turn, sumoylation of Scc1 was shown to counteract the action of Wapl, a negative regulator of cohesin loading (Wu and Yu 2012).

ChIP experiments in mouse B cells showed that SMC5 co-localizes with RPA, the single-strand binding protein involved in DNA replication and repair, and BRCA1, a protein involved in DSB repair, at early replication fragile sites (Barlow et al. 2013). These findings suggest that the SMC5/6 complex binds to single-stranded DNA (ssDNA) substrates created during HR and/or DNA replication.

### Regulation of homologous recombination in repetitive sequences

In budding yeast, the ribosomal genes are organized into a single array of 100–200 identical repeats on chromosome XII that is compartmentalized into the chromatin region called nucleolus (Oakes et al. 2006). Due to the repetitive nature of the ribosomal DNA (rDNA) locus, HR-mediated DNA damage repair in this region can lead to illegitimate recombination events that result in JMs and unequal sister chromatid exchange (Eckert-Boulet and Lisby 2009). In order to prevent such deleterious recombination events, DSBs occurring within rDNA are thought to be moved outside the nucleolus by a Smc5/6-dependent mechanism in order to be repaired (Torres-Rosell et al. 2005a, 2007). However, the visible presence of DSBs in the nucleolus of Smc5/6 mutants could also be due to less efficient repair of these breaks without functional Smc5/6.

Similarly, in *Drosophila*, Smc5/6 is thought to be involved in the translocation of the damaged DNA within heterochromatin regions to adjacent euchromatic regions where recombination can occur proficiently (Chiolo et al. 2011). Moreover, in heterochromatin, Smc5/6 suppresses HR until translocation of the DSB has occurred (Chiolo et al. 2011).

### Mitotic metaphase

Smc6 location in mitotic metaphase cells has been studied multiple times, with varying outcomes. Some studies in mouse and human show that SMC6 is translocated away from the chromosomes during mitotic divisions (Gallego-Paez et al. 2014; Taylor et al. 2001; Verver et al. 2013, 2014), while other studies in budding yeast and mouse report Smc6 to be located at the centromeres of mitotic cells (Gomez et al. 2013; Lindroos et al. 2006; Yong-Gonzales et al. 2012).

The SMC5/6 complex is required for regulating topoisomerase IIα and condensin localization on replicated chromatids in human cells during mitosis, thereby ensuring correct chromosome morphology and segregation (Gallego-Paez et al. 2014). Topoisomerase II (TopoII) resolves DNA topological constraints by introducing transient DSBs that are needed to decatenate double-stranded DNA to alleviate supercoiling (Nitiss 2009). TopoII initiates the passage of an unbroken DNA strand through the DSB and then reseals the break (Nitiss 2009). In budding yeast, Smc5/6 has recently been implicated in managing replication-induced topological stress (Carter and Sjogren 2012; Jeppsson et al. 2014a) and induction of topological stress by TopoII inactivation correlates with increased frequency of Smc5/6 chromosomal association sites (Jeppsson et al. 2014a; Kegel et al. 2011). In fission yeast, TopoII and Smc5/6 are required for the timely removal of cohesins from the chromosome arms before metaphase (Tapia-Alveal et al. 2010). Retention of these cohesins would otherwise cause chromosome missegregation and subsequent mitotic catastrophe. This was further supported when overexpression of separase, a protein that cleaves cohesin, was shown to rescue the lethality of TopoII and Smc5/6 mutants in fission yeast (Outwin et al. 2009).

## Meiosis

Meiosis is a specialized cell division during which one round of DNA replication is followed by two successive rounds of chromosome segregation. First, the homologous chromosomes, each consisting of one pair of sister chromatids held together by cohesin complexes, move to opposite poles (meiosis I). Second, the sister chromatids are segregated, resulting in the formation of four haploid cells (meiosis II). During prophase I, the homologous chromosomes align and, in most organisms, chromosome synapsis is achieved by formation of the synaptonemal complex (SC). Correct synapsis of the homologous chromosomes is required to facilitate meiotic recombination and the subsequent formation of meiotic crossovers. These meiotic crossovers, or chiasmata, introduce genetic variation among the resulting gametes. Additionally, together with proper sister chromatid cohesion, they also ensure correct chromosome orientation and segregation during meiosis I (reviewed in (Petronczki et al. 2003)).

**Fig. 2:**
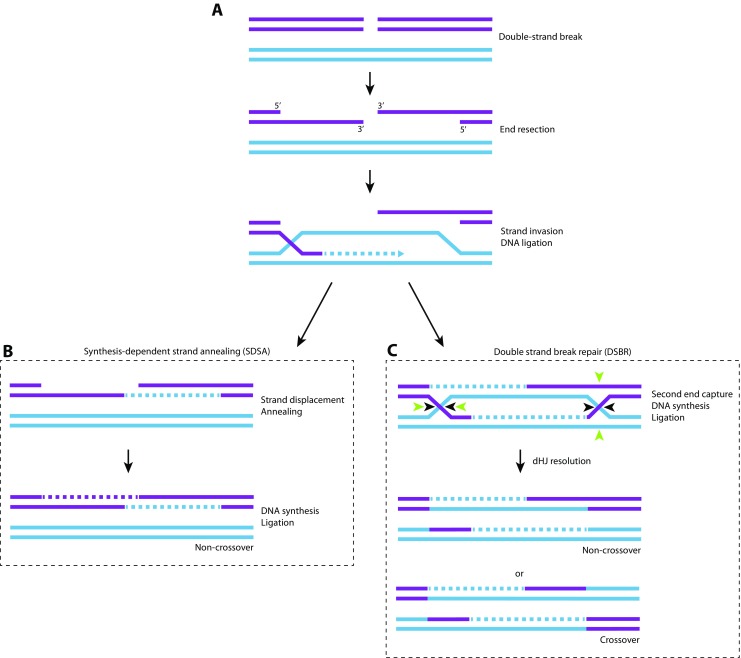
DNA double strand break repair by homologous recombination. **a** When a DNA double strand break (DSB) occurs, the DNA around the 5′ end is resected, creating a 3′ single-stranded DNA (ssDNA) overhang. This 3′ ssDNA overhang invades a homologous sequence, creating a D-loop. DNA is synthesized at the invading end using the undamaged template DNA strand. After this, further repair can be executed by synthesis-dependent strand annealing (SDSA) or double strand break repair (DSBR). **b** SDSA: The second DSB end will be annealed up to the ssDNA on the other break end, followed by gap-filling DNA synthesis and ligation. This will lead to a non-crossover event. **c** DSBR: The second DSB end can be captured to form a double Holliday Junction (HJ). The resulting recombination intermediate must be resolved by nicking the HJs. Depending on the nick sites, either parallel (*black arrows*) or anti-parallel (*green arrows)*, this will produce a non-crossover or a crossover event, respectively

The molecular pathways required for DSB repair during meiosis have been studied in most detail in budding yeast (De Muyt et al. 2012; Zakharyevich et al. 2012). However, evidence indicates that these pathways are conserved (Berchowitz et al. 2007; Higgins et al. 2008; Holloway et al. 2008). The following paragraphs briefly summarize meiotic recombination, using budding yeast as an example (Fig. 2). Meiotic recombination is initiated by Spo11-induced DSB formation, a 5-3′ exonuclease that produces a 3′ single-stranded DNA overhang at every break (Keeney et al. 1997). This 3′ overhang is then coated by the Rad51/Dmc1 strand exchange proteins and invades the complementary sequence of the homologous chromosome (Fig. 2a). DNA synthesis then starts from the invading end and proceeds beyond the DSB. This single-end invasion (SEI) is the precursor of all recombination pathways during meiosis (De Muyt et al. 2012; Zakharyevich et al. 2012).

Following SEI, most recombination events are processed via synthesis-dependent single-strand annealing (SDSA) (Fig. 2b). During SDSA, the invading strand is thought to be displaced by the RecQ helicase BLM/Sgs1 (Bennett et al. 1998; De Muyt et al. 2012; Jessop and Lichten 2008; Jessop et al. 2006; Oh et al. 2008). The displaced strand is then used as a synthesis template for the other damaged ssDNA end, and ligation results in the formation of a non-crossover.

The DSB repair mechanism in budding yeast that ensures reciprocal crossover formation is known as the ZMM (Zip1-4, Mlh1/3, Msh4/5) pathway. The ZMM pathway requires both SC components (Zip1-4 and Spo16) and the conserved mismatch repair heterodimers MutSγ (Msh4-5) and MutLγ (Mlh1-3) (Borner et al. 2004; Lynn et al. 2007). At a ZMM designated recombination site, the SEI is stabilized and the second end of the DSB is captured to form a double Holliday junction (dHJ). Interestingly, Sgs1 is required to stabilize the ZMM designated dHJs, which are resolved asymmetrically by Exo1-MutLγ to form COs, and eventually lead to chiasmata (Zakharyevich et al. 2012) (Fig. 2c).

Timely organization of the different steps of meiotic DSB repair depends on tight regulation of the meiotic prophase I, which can be subdivided in four stages: leptonema, zygonema, pachynema, and diplonema. During leptonema, the chromatin condenses and formation of axial elements between sister chromatids begin to form. Simultaneously, DSBs are induced by the endonuclease SPO11, triggering the meiotic DNA damage response. During zygonema, homologous chromosomes begin to synapse, characterized by the formation of the SC, a proteinaceous structure which comprises axial proteins (now termed lateral elements) linked by central components. Single-strand invasion occurs, followed by resection and DNA synthesis, resulting in recombination intermediates. Recombination events are neither randomly nor equally distributed throughout the genome but are preferentially located at hotspots at which DSBs are more frequently formed (reviewed in (Keeney et al. 2014)). At pachynema, the homologous chromosomes are fully synapsed along their entire length. DSB repair via HR continues by the resolution of recombination intermediates into either a non-crossover or a crossover event. Only a minority of recombination intermediates are resolved as crossovers, but there are processes which ensure that at least one crossover is formed per homolog pair (reviewed in (Youds and Boulton 2011)). Finally, in diplonema, the synaptonemal complex gradually dissociates and most recombination intermediates are completely resolved. Importantly, crossovers remain as chiasmata in order to keep homologous chromosomes locally tethered and, together with proper chromosome cohesion, ensure bi-orientation and accurate segregation during meiosis I (reviewed in (Petronczki et al. 2003)).

During the first meiotic division, homologous chromosomes, each containing two sister chromatids held together by cohesins, segregate to opposite poles. Bi-orientation of homologous chromosomes is crucial for their accurate segregation, and misalignment may result in aneuploidy. The spindle assembly checkpoint (SAC) controls this bi-orientation by monitoring the tension that is generated when the homologous chromosomes are pulled to opposite directions and only allows subsequent chromosome segregation when all chromosomes are correctly orientated. The physical linkage that chiasmata provide achieves bi-orientation and inter-homolog tension. Failure to generate the chiasmata, e.g., due to absence of DSB induction, inadequate repair, and lack of CO events, will lead to either a SAC induced metaphase I arrest and apoptosis or aberrant chromosome segregation and aneuploidy in the resulting gametes.

## Localization of Smc5/6 in meiosis

### Budding yeast

Using immunofluorescence microscopy, Smc6 was observed to localize to the nucleolus in budding yeast at the entry into meiosis (Farmer et al. 2011; Lilienthal et al. 2013). During meiotic progression, chromosome axes are formed and DSB repair is initiated. At this time, Smc5 and Smc6 localize as distinct foci along the chromosome axes (Copsey et al. 2013; Farmer et al. 2011; Lilienthal et al. 2013; Xaver et al. 2013). Smc6 also frequently co-localizes side by side with Rad51 recombinase, indicating a potential function in the strand invasion step in HR repair (Copsey et al. 2013; Xaver et al. 2013). The Smc6 localization along the axes becomes more abundant as synapsis occurs (Copsey et al. 2013; Lilienthal et al. 2013; Xaver et al. 2013). The formation of this punctate distribution does not depend on meiotic DSBs (Copsey et al. 2013; Farmer et al. 2011). Contrasting data has been reported for the effect of cohesin mutation on Smc5/6 axis loading. It was observed by Lillienthal et al. that Smc6 binding to chromosomes is dependent on meiosis-specific cohesin subunit Rec8. Therefore, the Smc5/6 complex may be influenced by meiotic axis structure and/or the presence of sister chromatid cohesion. In contrast, however, the localization of Smc5 was not affected by the absence of Rec8 (Copsey et al. 2013). Although surprising, it is possible that Smc5 and Smc6 loading to chromosome axes is independent of one another, and Smc6 but not Smc5 requires cohesin. An alternative explanation is that differences in chromatin spreading techniques resulted in the contrasting observations. The localization of Smc5/6 during late prophase is still inconclusive. After late prophase, some studies reported that Smc5 and Smc6 localization become more diffuse and are absent prior to metaphase I (Copsey et al. 2013; Lilienthal et al. 2013), while another study reported that Smc6 localized to the chromatin during both meiotic divisions, displaying dense clusters at the boundary between segregating chromatin masses (Xaver et al. 2013). These discrepancies may be due to sensitivity differences in chromatin spreading technique and epitope accessibility.

To assess chromatin localization of Smc5/6 in greater detail, genome-wide ChIP-on-chip localization studies were used (Copsey et al. 2013; Xaver et al. 2013). These studies showed that Smc5 and Smc6 bind to many of the same chromosomal axis-associated sites as Rec8, including centromeres. In addition, Smc5/6 is enriched at DSB hotspots. However, this localization occurs independently of DSB formation, which supports the immunofluorescence microscopy data (Copsey et al. 2013; Xaver et al. 2013). Finally, as observed in mitotic cells, Smc5/6 also binds to the rDNA, which remains unsynapsed during meiotic prophase I (Farmer et al. 2011; Lilienthal et al. 2013; Xaver et al. 2013).

### *Caenorhabditis elegans*

In *C. elegans*, SMC-6 localizes to the condensed chromatin of germ cells throughout meiosis (Bickel et al. 2010). SMC-6 becomes enriched on chromosomes during pachytene, which coincides with occurrence of DSB repair, complementing the localization pattern in budding yeast. SMC-6 remains on chromosome axes during diplotene and diakinesis in worms (Bickel et al. 2010).

### Mouse, human

The first indications of a possible role for SMC5/6 in mammalian meiotic progression were elevated levels of both SMC5 and SMC6 in the testis and localization in spermatocytes (Taylor et al. 2001). It then took over 12 years before the role of SMC5/6 in mammalian meiosis was investigated in more depth revealing involvement at several crucial and diverse steps during rodent and human spermatogenesis (Gomez et al. 2013; Verver et al. 2013, 2014). First, in mouse spermatocytes, SMC5, SMC6, and NSMCE1 were found to be located at pericentromeric heterochromatin (or so-called chromocenters): condensed repetitive sequences surrounding the centromeres (Gomez et al. 2013; Verver et al. 2013). This localization already starts in differentiating spermatogonia, remains throughout all meiotic stages, including metaphase I and II, and disappears when the haploid spermatids start to elongate (Gomez et al. 2013; Verver et al. 2013). Moreover, SMC5 and SMC6 were detected at the SC of synapsed homologous chromosomes from early zygonema until late diplonema in mouse spermatocytes (Gomez et al. 2013). This latter localization pattern was also reported for both SMC5 and SMC6 in human spermatocytes (Verver et al. 2014). Finally, detection of SMC5, SMC6, and NSMCE1 at the XY body during pachynema was observed in both mouse (Gomez et al. 2013; Taylor et al. 2001) and human spermatocytes (Verver et al. 2014). However, it must be noted that in mouse spermatocytes, SMC5, SMC6, and NSMCE1 localize to the chromatin of the XY body (Gomez et al. 2013), whereas in human spermatocytes, the localization of SMC6 was limited to distinct foci located at the axial elements of the unsynapsed X and Y chromosomes (Verver et al. 2014).

## Functions of Smc5/6 in meiosis

### Meiotic recombination

When meiotic recombination intermediates are not properly resolved to form either a non-crossover or crossover, aberrant joint molecules (JMs) can emerge. These JMs have the potential to block chromosome segregation if unresolved (Copsey et al. 2013; Jessop and Lichten 2008; Xaver et al. 2013). Sgs1 limits the formation of these JM structures (Chen et al. 2010; De Muyt et al. 2012; Fabre et al. 2002; Jessop and Lichten 2008; Sugawara et al. 2004). Several structure-selective nucleases, Mus81-Mms4, Slx1-Slx4, and Yen1, are involved in the resolution in these JMs (De Muyt et al. 2012; Matos et al. 2011; Zakharyevich et al. 2012). In budding yeast, the Smc5/6 complex antagonizes the formation of JMs via two mechanisms: (i) prevention of JMs by destabilizing SEI intermediates (Xaver et al. 2013) and (ii) facilitating JM resolution (Copsey et al. 2013; Lilienthal et al. 2013; Xaver et al. 2013). Like previously reported for the helicase BLM/Sgs1, the SUMO E3 ligase function of Nse2/Mms21 subunit is required to destabilize SEI intermediates (Xaver et al. 2013). This inhibition is needed to prevent the formation of inappropriate recombination intermediates. In the absence of Smc5/6, these inappropriate recombination intermediates develop into JMs that require the structure-selective resolvases Mus81-Mms4, Slx1-Slx4, and Yen1 to be processed (Zakharyevich et al. 2012). Of these resolvases, at least the ability of Mus81 to associate with, or be stabilized on, the meiotic chromosomes efficiently is dependent on Smc5/6 (Copsey et al. 2013). Interestingly, while required to limit SEI stabilization, the SUMO E3 ligase function of Nse2/Mms21 is not required for Smc5/6 directed JM resolution (Xaver et al. 2013).

In fission yeast, meiotic recombination generates single Holliday junction (HJ) intermediates (Cromie et al. 2006; Davis and Smith 2003; Hyppa and Smith 2010; Keeney et al. 1997), which are eventually resolved by the Mus81-Eme1 complex (Boddy et al. 2001; Cromie et al. 2006; Osman et al. 2003). Based on genetic experiments, the Smc5/6 complex subunits Nse5-Nse6 have a regulatory role in Mus81-Eme1 dependent HJ resolution (Wehrkamp-Richter et al. 2012).

In *C. elegans*, the SMC-5/6 complex is not required for chiasmata formation. However, mutation of *smc*-*5* or *smc*-*6* did result in chromosome fragmentation during meiosis I and an increased number of RAD-51 foci in the nucleus (Bickel et al. 2010). Interestingly, *mus*-*81*, *him*-*6* (a BLM ortholog), and *mus*-*81*, *xpf*-*1* double mutants display a similar phenotype to the *smc*-*5* or *smc*-*6* mutants (O’Neil et al. 2013). Because these genes are involved in two redundant HJ resolution pathways in *C. elegans* (Agostinho et al. 2013), the SMC-5/6 complex is likely to be involved in HJ resolution. Hence, the *C. elegans* SMC-5/6 complex may be playing similar JM antagonistic roles observed in budding yeast by hindering JM formation early and assisting JM resolution. However, *C. elegans* chromosomes are holocentric, and subsequent roles of SMC-5/6 in chromosome segregation may differ from other model organisms.

### Preventing HR in heterochromatin

In budding yeast, Smc5/6 also binds to the rDNA, which remains unsynapsed during meiotic prophase I (Farmer et al. 2011; Lilienthal et al. 2013; Xaver et al. 2013). Smc5/6 has been shown to have an anti-recombinogenic role at this repetitive DNA locus during vegetative growth (Torres-Rosell et al. 2007). Additionally, budding yeast Smc6 is strongly enriched in the pericentromeric regions during the mitotic G2 phase (Lindroos et al. 2006). Smc5/6 is essential for the timely separation of chromatids and the prevention of branched and entangled chromosome structures and subsequent mitotic arrest (Lindroos et al. 2006). It is conceivable that Smc5/6 plays similar roles at the rDNA locus and pericentromeric regions during meiosis.

In mouse spermatocytes, SMC5, SMC6, and NSMCE1 localize at pericentromeric heterochromatin (Gomez et al. 2013; Verver et al. 2013). As with rDNA, these regions are at high risk of aberrant recombination events when HR is enabled, leading to genomic instability (Goodarzi and Jeggo 2012). An additional challenge specific to meiotic cells is the endogenous induction of DSBs that are repaired by HR. Pericentromeric heterochromatin consists of densely packed repetitive sequences and is therefore vulnerable to aberrant events such as the formation of intra-chromosomal recombination structures. As a result, meiotic recombination is generally suppressed around the centromeres (Lynn et al. 2004), via a mechanism yet to be elucidated. The role of Smc6 in preventing HR in these high-risk regions has already been established for yeast and *Drosophila* mitotic cells (Chiolo et al. 2011; Torres-Rosell et al. 2007). In line with these studies, pericentromeric heterochromatin of mouse prophase spermatocytes is simultaneously marked with SMC5, SMC6, and NSMCE1 (Gomez et al. 2013; Verver et al. 2013) and deprived of recombination sites marked by RAD51 (Verver et al. 2013). These findings suggest that also in mammalian germ cells, SMC5/6 might be responsible for preventing aberrant HR events in repetitive sequences. Interestingly, even though prevention of HR in heterochromatin might be a conserved function of SMC5/6, a similar localization was not found in human prophase spermatocytes (Verver et al. 2014).

### Centromere cohesion

During budding yeast meiosis, Smc5/6 regulates sister chromatid cohesion at centromeres and is required for the timely removal of cohesin from chromosomal arms (Copsey et al. 2013).

SMC6 is proximal to the centromeres during both meiotic metaphases in mouse (Gomez et al. 2013; Verver et al. 2013) and human (Verver et al. 2014). As well as during prophase I stages, SMC6 co-localizes at the centromeres with Topo IIα during metaphase I and II (Gomez et al. 2013). More specifically, in metaphase I and anaphase I, SMC6 was present as two foci proximal to the sister kinetochores, and only one signal near the kinetochores at metaphase II and anaphase II (Gomez et al. 2013). Additionally, in metaphase II spermatocytes, in which the centromeres are subjected to tension from opposite poles, SMC6 appeared as a strand connecting the sister kinetochores (Gomez et al. 2013). The finding that SMC6 co-localizes with Topo IIα, together with the fact that the strand of SMC6 joining sister kinetochores persists even after redistribution of Aurora-B, suggests that the SMC5/6 complex may regulate sister chromatid centromere cohesion and dissolution of DNA catenates that form after DNA replication (Gomez et al. 2013). This role for SMC5/6 was further appointed when Topo IIα was inhibited by etoposide, inducing lagging chromosomes during the second meiotic division. Both SMC6 and Topo IIα co-localized at stretched strands connecting these lagging chromatids at the site of the kinetochores (Gomez et al. 2013). Complementary data was acquired using budding yeast, where localization of Smc5 depends on meiotic DNA replication, and in the absence of TopoII, Smc5 localization is aberrant (Copsey et al. 2013).

### SC assembly/stability, homologous chromosome synapsis

Both in mouse and human spermatocytes, SMC5 and SMC6 were found to be located at the SC (Gomez et al. 2013; Verver et al. 2014). Co-localization of mouse SMC6 with the SC central region proteins SYCP1 and TEX12 showed that SMC6 is restricted to synapsed chromosomes, leaving the un- or desynapsed axes including X and Y, unmarked (Gomez et al. 2013). Mammalian synapsis is characterized by the presence of a central region that, besides SYCP1 (equivalent to Zip1 in budding yeast), also contains the central element proteins SYCE1-3 and TEX12 (Bolcun-Filas et al. 2007, 2009; Hamer et al. 2006, 2008; Schramm et al. 2011). However, although dependent on SYCP1, loading of SMC6 to the mouse SC occurs independent of these central element proteins (Gomez et al. 2013). Additionally, mouse SMC5/6 localization is not dependent on meiosis-specific cohesin subunits REC8 and SMC1β (Gomez et al. 2013). The longitudinal localization pattern along the mammalian synapsed SC axes could suggest that localization of SMC5/6 is dictated by chromosome structure, as has been suggested in mitotic cells (Jeppsson et al. 2014b), or that the complex either facilitates SC assembly, chromosome synapsis, or recruitment of other SC-associated proteins.

### The XY body and unsynapsed chromosomes in pachytene spermatocytes

In males, due to a lack of homology, the X and Y chromosomes remain largely unsynapsed during the meiotic prophase I. During meiotic prophase, unsynapsed chromosomal regions are transcriptionally silenced by a process called meiotic silencing of unsynapsed chromosomes (MSUC) (Ichijima et al. 2012). In the case of the X and Y chromosome, this silencing is called meiotic sex chromosome inactivation (MSCI), and is achieved by the formation of a so-called XY body (or sex body), marked by the presence of several DNA damage response proteins such as BRCA1, γ-H2AX, and ATR (Ichijima et al. 2012). In male meiotic cells with extensive autosomal asynapsis, MSUC competes with MSCI for these proteins. The sex chromosomes will then be inadequately silenced, which will result in a pachytene arrest (Burgoyne et al. 2009). In mouse spermatocytes, SMC5, SMC6, and NSMCE1 were found to cover the XY body (Gomez et al. 2013). Because the XY staining resembles that of γ-H2AX, it is proposed that the SMC5/6 complex might be facilitating MSCI at this site.

In human spermatocytes, SMC6 is present on the unsynapsed XY chromosomes in a more foci-like pattern (Verver et al. 2014), suggesting a function in DSB repair. Interestingly, it has been recently found that in the absence of synapsis, including the unsynapsed regions of the sex chromosomes, SPO11 will continue to make DSBs (Kauppi et al. 2013). In this light, the presence of SMC5/6 on the unsynapsed sex chromosomes might be required to repair these continuously induced DSBs. In addition to this observation, unsynapsed autosomes display both RAD51 and SMC6 foci (Verver et al. 2014). Hence, it seems likely that human SMC5/6 plays a role in the repair of the continuously induced DSBs on unsynapsed meiotic chromosomes.

## Discussion/concluding remarks

**Fig. 3:**
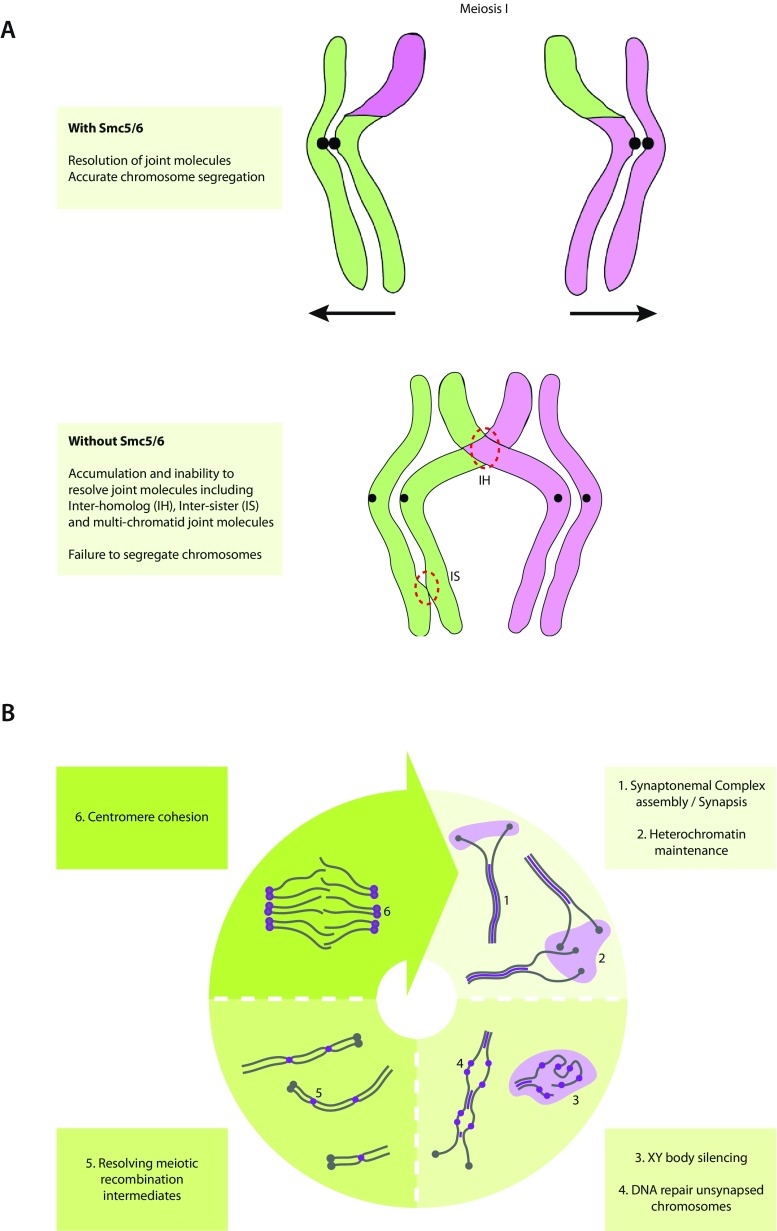
Proposed functions of Smc5/6 in meiosis. **a** In budding and fission yeast, Smc5/6 is required for the resolution of meiotically induced joint molecules and correct segregation of homologous chromosomes. Without functional Smc5/6 recombination intermediates cannot be efficiently resolved, leading to the accumulation of inter-homolog, inter-sister, and multi-chromatid joint molecules and failure to segregate chromosomes properly. *Black spot* = centromere. **b** During mouse and human meiosis, SMC5/6 functions in a variety of processes. It is proposed to be involved in synaptonemal complex formation and/or stability, heterochromatin maintenance, and XY body silencing. Moreover, it may be required for repair of DSBs due to lack of synapsis and resolving meiotic recombination intermediates. Finally, SMC5/6 is involved in centromere cohesion during M-phase. *Purple* = SMC5/6 complex localization. *Gray filaments* = lateral elements of the synaptonemal complex. *Gray spot* = centromere. Note: depicted chromosomes represent (telocentric) mouse chromosomes

In recent years, assessment of Smc5/6 localization and analysis of Smc5/6 mutant phenotypes during meiosis has resulted in an abundance of data implying a number of meiotic functions (Table 1). In all models, and in line with its described functions during mitosis, Smc5/6 is involved in HR-mediated repair and chromosome segregation, as depicted in Fig. 3. However, despite this common denominator, the meiotic functions of Smc5/6 seem astonishingly diverse.

**Table 1:** Proposed functions of Smc5/6 in meiosis Smc5/6 function in meiosis has been studied in different model organisms, leading to different proposed roles. This table lists the results leading to the allocation of a role for Smc5/6 in a certain meiotic process

Organism	Meiotic function					
	Response to DSBs	Meiotic recombination	Heterochromatin maintenance	Centromere cohesion	Homologous chromosome synapsis	Meiotic sex chromosome inactivation
Budding Yeast *S. Cerevisiae*	- Co-localization of Smc6 side by side with Rad51 (Copsey et al. 2013; Xaver et al. 2013) - Smc5/6 enriched at DSB hotspots (Copsey et al. 2013; Xaver et al. 2013)	- Smc5/6 antagonizes the formation of joint molecules (Copsey et al. 2013; Lilienthal et al. 2013; Xaver et al. 2013) - Smc5/6 is required for regular localization of Mus81 (Copsey et al. 2013; Xaver et al. 2013)	- Localization of Smc5/6 to rDNA (Farmer et al. 2011; Lilienthal et al. 2013; Xaver et al. 2013). May have similar anti-recombinogenic role at rDNA as observed during vegetative growth (Torres-Rosell et al. 2007)	- Smc5/6 regulates centromere cohesion and required for timely removal of cohesin from chromosomal arms (Copsey et al. 2013)	- Localization of Smc5 and Smc6 along synapsed axes (Copsey et al. 2013; Lilienthal et al. 2013; Xaver et al. 2013)	
Fission Yeast *S. Pombe*	- Nse6 acts after the Rad51 and Dmc1 strand exchange proteins (Wehrkamp-Richter et al. 2012)	- Regulatory role of Nse5-Nse6 in Mus81-Eme1 dependent Holliday junction resolution (Wehrkamp-Richter et al. 2012)				
Worm *C. elegans*	- Enrichment of SMC-6 with the occurrence of DSB repair (Bickel et al. 2010) - Chromosome fragmentation and increased number of RAD-51 foci during MI in *smc*-*5* or *smc*-*6* (Bickel et al. 2010)	- Mutations *smc*-*5* or *smc*-*6* and double mutants *mus*-*81*, *him*-*6*, and *mus81*, xpf-1 display similar phenotypes (Agostinho et al. 2013; O’Neil et al. 2013)			- Enrichment of SMC-5 and SMC-6 on chromosome axes during pachytene (Bickel et al. 2010)	
Mouse *M. Musculus*			- Localization of SMC5, SMC6, and NSMCE1 to pericentromeric heterochromatin (Gomez et al. 2013; Verver et al. 2013), which is simultaneously deprived of HR (Verver et al. 2013)	- Localization of SMC6 to centromeres (Gomez et al. 2013; Verver et al. 2013) - Co-localization of SMC6 with Topo IIα (Gomez et al. 2013)	- Localization of SMC5 and SMC6 on synapsed axes, co-localization with SYCP1 and TEX12 (Gomez et al. 2013)	- Localization of SMC5 and SMC6 to the XY body, similar pattern as γ-H2AX (Gomez et al. 2013; Verver et al. 2013)
Human *H. Sapiens*	- Localization of SMC6 foci to unsynapsed chromosomes, side by side to RAD51 foci (Verver et al. 2014)			- Localization of SMC6 to centromeres (Verver et al. 2014)	- Localization of SMC5 and SMC6 to synapsed axes (Verver et al. 2014)	- Localization of SMC6 foci to the XY chromosome axes (Verver et al. 2014)

Several studies in mammalian models have shown varying results when using antibodies against different epitopes of SMC6 simultaneously (Gomez et al. 2013; Verver et al. 2013, 2014). Since the technical variation within experiments was negligible, differences in localization pattern are most likely a reflection of varying conformations of the SMC6 protein or SMC5/6 complex as a whole, resulting in differing accessibility of these epitopes. Indeed, a study using budding yeast demonstrates that the Smc5/6 complex is physically remodeled in an ATP-dependent manner (Bermudez-Lopez et al. 2015). Even though future studies might unravel the role of conformation herein, another possibility is that Smc6 and/or Smc5 can act independently from the Smc5/6 complex, thereby showing differential localization patterns. When budding yeast proteins were purified separately, Smc5 and Smc6 were found to have some binding activity to ssDNA, independently of the presence of the other subunits (Roy and D’Amours 2011; Roy et al. 2011). However, even though some studies support the complex-independent function of Smc5 and Smc6 (Laflamme et al. 2014; Roy et al. 2011; Vignard et al. 2011), most studies show that hypomorphic alleles and RNAi knockdown of Smc5 and Smc6 yield complementary phenotypes (e.g., (Gallego-Paez et al. 2014; Torres-Rosell et al. 2005b)). Moreover, fractionation experiments indicate that the majority of Smc5/6 components are in complex, and only a small fraction is present as isolated monomers (Torres-Rosell and Losada 2011).

The diversity of mitotic and meiotic functions of Smc5/6 illustrates the versatility of this protein complex. Yet, the regions where Smc5/6 has been found to act are not random and its roles on rDNA, telomeres, DSBs, replication sites, and collapsed replication forks all support the strong preference of Smc5 and Smc6 to capture ssDNA (Roy and D’Amours 2011; Roy et al. 2011). The consequences of Smc5/6 binding to DNA seem to vary between the specific processes it is required for. During meiosis, the Smc5/6 complex can either link homologous chromosomes or may recruit other proteins to its site of action. Either way, both the fission and budding yeast Smc5/6 complex have been found to be crucial to resolve meiotic recombination intermediates (Copsey et al. 2013; Lilienthal et al. 2013; Xaver et al. 2013). Despite its seemingly diverse roles during different processes, resolving complex chromosome structures, which would otherwise cause cell cycle arrest or prevent chromosomes from being segregated, appears a major meiotic function of Smc5/6. However, how Smc5/6 is molecularly regulated during different meiotic processes, such as pre-meiotic S-phase, meiotic recombination, and the meiotic M-phases, still needs further research. Creation and assessment of mammalian mutant models, together with the development of a comprehensive meiosis interactome for the SMC5/6 complex will further our comprehension of SMC5/6 functions. More knowledge on the meiotic functions of Smc5/6 may give insight in one of the biggest questions in biology: how are germ cells capable to passage their genome through essentially endless generations while maintaining sufficient genomic integrity.
